# 

**Published:** 1880-05-20

**Authors:** 


### EDITORIAL AND MISCELLANEOUS.

☞ **Please Read.**—This number of the RECORD will be sent to certain brethren whose names we dropped from the roll in January, for non-payment.

Friends, we regretted to do it, but the draught was too heavy upon us. As sensible men you cannot but feel that we did not desert you, but you deserted us. We suppose you are clever men but careless, but the injury and loss to us was all the same.

Will these brethren not pay up back dues and renew their subscriptions?

---

☞ We receipt all by letter or postal card who request it, but prefer to receipt in the Journal to save postage. We beg to say to those who send by registered letter or postal order, that a special receipt is not necessary, as the post office records will always establish the fact if a question arises as to a remittance having been made.

---

*Cinchonia.*—We invite special attention to the advertisement of this article in to-day's Journal, by Messrs. Powers & Weightman.

*Reynolds' System of Medicine.*—In our notice of this valuable work. in our March No., we should have stated for the benefit of our readers that this is the same work as that published in England in five volumes, and sold at a very high price.

The five volumes and five thousand pages of the original is, by the use of a condensed but very clear type, and double columns, compressed into *three* volumes of about three thousand pages, which includes the American additions by Henry Hartshorne, M. D.

Price, $5.00 per volume, cloth.

The work is sold by subscripiion. Messrs. J. H. Chambers & Co., Atlanta, Ga., are the general agents for the South.

*Donation to the College Museum.*—Dr. W. S. Morgan, of Houston, Ga., has presented to the Museum of the *Southern Medical College* a very interesting and remarkable specimen of abnormal fœtation. We hope to procure from the Doctor a history of the case, before describing it in detail. He has the thanks of the Faculty for the valuable donation.

*New Journals.*—The following letter contains, we think, a useful hint which we hope our readers will profit by :

EDITOR OF RECORD :—*Dear Sir:* We dropped your Journal two years ago to take one which we thought equally as good, and cheaper. But we confess that we have been disappointed, and we return to our first love.

Enclosed find $6.00 for which please send us the two back volumes we have lost, and renew our subscription to our old friend, the Record.

